# Lokomat-Assisted Robotic Rehabilitation in Spinal Cord Injury: A Biomechanical and Machine Learning Evaluation of Functional Symmetry and Predictive Factors

**DOI:** 10.3390/bioengineering12070752

**Published:** 2025-07-10

**Authors:** Alexandru Bogdan Ilies, Cornel Cheregi, Hassan Hassan Thowayeb, Jan Reinald Wendt, Maur Sebastian Horgos, Liviu Lazar

**Affiliations:** 1 Faculty of Medicine and Pharmacy, University of Oradea, Str. Piata 1 Decembrie nr. 10, 410087 Oradea, Romania; ilies.alexb@yahoo.com (A.B.I.); cornel.cheregi@yahoo.com (C.C.); lazarlv@yahoo.com (L.L.); 2 Social Studies Department, College of Arts, King Faisal University, Al-Ahsa 31982, Saudi Arabia; 3 Szpital Św. Wincentego a Paulo w Gdyni, ul. Wójta Radtkego 1, 81-348 Gdynia, Poland; wendtjr@gmail.com

**Keywords:** spinal cord injury, Lokomat, robotic rehabilitation, biomechanical symmetry, machine learning, gait training, L-ROM, L-STIFF, L-FORCE, predictive modeling

## Abstract

Background: Lokomat-assisted robotic rehabilitation is increasingly used for gait restoration in patients with spinal cord injury (SCI). However, the objective evaluation of treatment effectiveness through biomechanical parameters and machine learning approaches remains underexplored. Methods: This study analyzed data from 29 SCI patients undergoing Lokomat-based rehabilitation. A dataset of 46 variables including range of motion (L-ROM), joint stiffness (L-STIFF), and muscular force (L-FORCE) was examined using statistical methods (paired *t*-test, ANOVA, and ordinary least squares regression), clustering techniques (k-means), dimensionality reduction (t-SNE), and anomaly detection (Isolation Forest). Predictive modeling was applied to assess the influence of age, speed, body weight, body weight support, and exercise duration on biomechanical outcomes. Results: No statistically significant asymmetries were found between left and right limb measurements, indicating functional symmetry post-treatment (*p* > 0.05). Clustering analysis revealed a weak structure among patient groups (Silhouette score ≈ 0.31). Isolation Forest identified minimal anomalies in stiffness data, supporting treatment consistency. Regression models showed that body weight and body weight support significantly influenced joint stiffness (*p* < 0.01), explaining up to 60% of the variance in outcomes. Conclusions: Lokomat-assisted robotic rehabilitation demonstrates high functional symmetry and biomechanical consistency in SCI patients. Machine learning methods provided meaningful insight into the structure and predictability of outcomes, highlighting the clinical value of weight and support parameters in tailoring recovery protocols.

## 1. Introduction

Spinal cord injury (SCI), whether of traumatic or non-traumatic origin, frequently leads to severe gait impairment, especially in individuals classified under ASIA Impairment Scale grades C and D which denote incomplete motor or sensory loss [1]. Walking is often possible in such patients but is significantly limited by poor coordination, asymmetric lower-limb function, and decreased balance [2]. Robotic-assisted rehabilitation has emerged as a promising approach to restore locomotor abilities through repetitive, physiological gait training. This strategy is employed not only in SCI but also in other neurological conditions, including stroke, traumatic brain injury, multiple sclerosis, and cerebral palsy [3,4,5]. The Lokomat system is one of the most widely used robotic gait orthoses. It combines body weight support, robotic actuators, and virtual reality feedback to facilitate structured and individualized gait therapy [6]. Patients undergoing Lokomat-assisted therapy are suspended via a harness, partially supporting their body weight, while robotic actuators guide their legs through predefined gait cycles. Visual feedback from integrated monitors enhances patient engagement and helps track progress [7]. Assistance may be tailored to patient needs through robotic movement control, physiotherapist intervention, or neuromuscular electrical stimulation, optimizing rehabilitation outcomes [8]. Holistic approaches to rehabilitation have increasingly considered systemic and environmental factors affecting patient recovery. Previous interdisciplinary investigations have highlighted how medical conditions during pregnancy or ecological quality can influence physiological outcomes, supporting the integration of biomechanical and clinical data in personalized therapeutic strategies [9,10,11]. The neuroplastic benefits of robotic rehabilitation are enhanced when combined with adjunctive pharmacological or neurostimulation strategies, promoting motor learning, functional independence, and reintegration into daily life [9,10,11,12,13]. Lokomat-based therapy has improved biomechanical symmetry, muscular coordination, and psychological and quality-of-life outcomes in individuals with partial gait impairments [14,15,16].

Therefore, this study aimed to evaluate the effectiveness of Lokomat-assisted robotic rehabilitation in patients with spinal cord injury by combining biomechanical analysis with machine learning techniques. Specifically, the research focused on assessing functional symmetry (L-ROM, L-FORCE, and L-STIFF) and identifying predictive clinical- and training-related factors influencing recovery outcomes. By integrating statistical modeling with clustering and anomaly detection methods, the study seeks to contribute novel insights into the personalization and optimization of robotic gait therapy.

## 2. Materials and Methods

Descriptive statistics were compiled for a cohort of 29 patients with spinal cord injury (SCI) undergoing rehabilitation at Baile Felix Recovery Hospital, Romania, using the Lokomat robotic system [1,2]. This system, extensively used for gait training, combines robotic actuators with a treadmill to assist hip and knee movements through four servo motors, aiming to restore walking ability after trauma and improve quality of life [3,4,5].

A total of 46 variables were analyzed, including independent factors such as age, distance walked in the simulator, duration of exercise, walking speed, and body weight support, alongside dependent variables that evaluated lower-limb function. The functional outcome measures included the following: Lower Range of Motion (L-ROM) in hip and knee flexion/extension (left and right limb); Lower Stiffness (L-STIFF) of hip and knee joints at three angular velocities (for both sides); and Lower Force (L-FORCE) for hip and knee joints (left and right limb).

L-ROM was measured passively, without robotic assistance, by evaluating resistive torque during joint flexion/extension at a constant velocity. Data analysis was conducted using JupyterLab (v4.1.5), Stata (v17.0 SE), and IBM SPSS Statistics (v28).

Asymmetries in hip and knee L-ROMs were assessed via paired *t*-tests. ANOVA tests revealed three distinct patient clusters. K-means clustering further examined groupings using independent variables (age, weight, body weight support, speed, distance, and duration). Cluster centroids were calculated using Euclidean distances and visualized in scatterplots.

K-means clustering is an unsupervised learning technique designed to organize a collection of *n* data points (×1, ×2, ..., ×n), each represented as a real-valued vector in a d-dimensional space, into k distinct groups or clusters (k ≤ *n*). The central goal of this method is to assign each data point to one of the k clusters so that the variability within each cluster is minimized. This is formally expressed by minimizing the within-cluster sum of squares (WCSS), quantifying the total squared distance between each data point and the centroid of the cluster to which it belongs. By performing this, k-means seeks to ensure that points within the same cluster are as similar as possible while also maintaining distinct separation between clusters.

The quality of clustering was determined using the Silhouette method; the Silhouette coefficients were calculated using Formula (1), which is as follows [7]:

(1) $$s\left(i\right)=\frac{b\left(i\right)-a(i)}{max\{a\left(i\right),\;b\left(i\right)\}},\;if\;\left|C_{I}\right|>I\;\;\;\;\;\;\;\;$$

where a(i) is the mean intra-cluster distance, and b(i) is the mean nearest-cluster distance [7].

Dimensionality reduction in L-FORCE variables was performed via t-distributed Stochastic Neighbor Embedding (t-SNE), a nonlinear visualization method, coupled with k-means clustering [8]. t-SNE models local similarity in high-dimensional data and minimizes divergence between original and low-dimensional space using Kullback–Leibler divergence [9].

The Isolation Forest algorithm was applied to identify anomalies in L-STIFF data. It isolates data points using binary trees (iTrees) built through random partitioning, with anomalous values more quickly isolated [10]. Anomaly scores were computed using average path lengths and harmonic number approximations.

**Figure 1:**
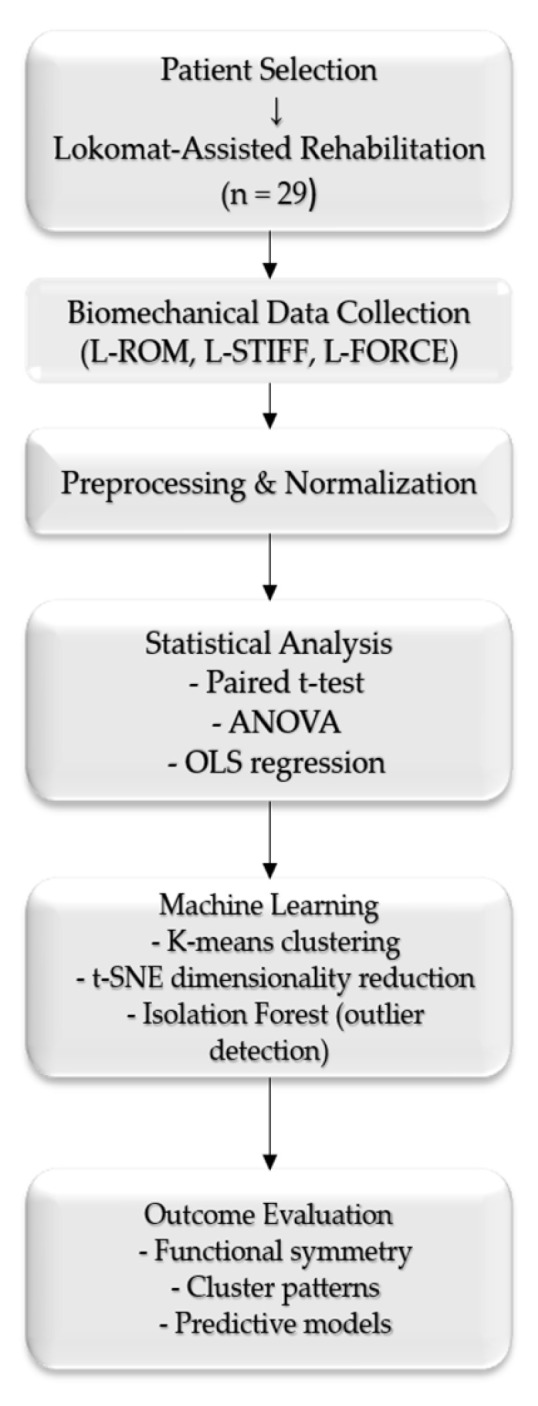
Overview of the study design and analysis pipeline, including patient selection, biomechanical data collection, statistical modeling, and machine learning methods.

Figure 1 presents a workflow summarizing the methodological steps to illustrate the study design and analytical sequence better.

### 2.1. t-SNE and K-Means Clustering for L-FORCE Analysis

The left force (L-FORCE) parameters, representing biomechanical performance in the hip and knee joints, were analyzed using t-distributed Stochastic Neighbor Embedding (t-SNE). This nonlinear dimensionality reduction technique is widely utilized for visualizing complex, high-dimensional data structures. T-SNE was combined with the k-means clustering algorithm to facilitate cluster formation and improve interpretability. K-means is a partitioning method that groups data into distinct clusters by minimizing intra-cluster variance. The integration of these two methods facilitated the projection of high-dimensional L-FORCE data onto a low-dimensional (two-dimensional or three-dimensional) space, allowing for the visualization of underlying patterns and hidden cluster structures within the dataset [14].

The t-SNE algorithm performs this transformation in two key stages. Initially, it computes a probability distribution over pairs of data points in the original high-dimensional space, assigning higher probabilities to similar points. Subsequently, it defines an equivalent distribution in the reduced space and minimizes the Kullback–Leibler (KL) divergence between the two. This ensures that local relationships in the original data are preserved in the visualization, allowing for meaningful grouping and separation of biomechanical profiles [15].

Clustering performance was evaluated using the Silhouette score and within-cluster sum of squares (WCSS). Silhouette score measures how similar an object is to its cluster compared to other clusters, ranging from −1 (poor fit) to + 1 (ideal fit). WCSS indicates the compactness of clusters.

**Figure 2:**
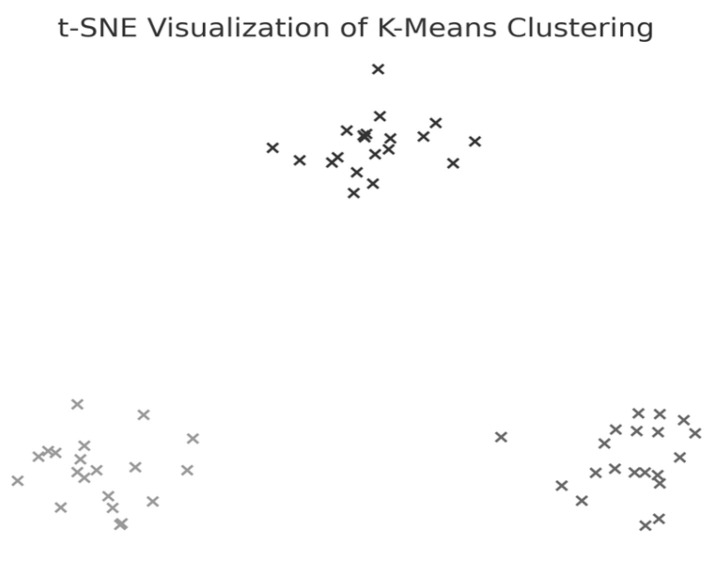
t-SNE visualization of k-means clustering applied to the rehabilitation dataset. Each point represents a subject’s biomechanical profile projected in a two-dimensional space, showing clear cluster separation.

The average Silhouette score was low (0.31), indicating a weak clustering structure and limited cohesion among subgroups. Nevertheless, k = 3 was selected for exploratory purposes as the Elbow method applied to the within-cluster sum of squares (WCSS) suggested limited improvement beyond this value. A t-distributed Stochastic Neighbor Embedding (t-SNE) representation was generated (Figure 2) to explore potential grouping patterns in two-dimensional space visually.

### 2.2. Anomaly Detection in L-STIFF Using Isolation Forest

The Isolation Forest algorithm was applied to detect potential outliers within the left stiffness (L-STIFF) dataset. This unsupervised learning technique is particularly effective for identifying anomalies in high-dimensional biomedical data. The core principle is based on the observation that anomalies are more susceptible to isolation because they differ significantly from most data points. Isolation Forest constructs multiple binary decision trees (iTrees) by recursively selecting features and split values at random. Data points isolated with fewer splits, i.e., shorter average path lengths, are flagged as anomalous. The anomaly score for each point is thus derived from its average path length across the ensemble of trees. This model is advantageous in rehabilitation data analysis, where subtle biomechanical deviations can indicate critical recovery issues [16]. This model is as follows:

(2) $$c\left(m\right)=\left\{\begin{array}{c} 2H\left(m-1\right)-\frac{2\left(m-1\right)}{n}\;for\;\;m>2\\ \;1\;\;\;\;\;\;\;\;\;\;\;\;\;\;\;\;\;\;\;\;\;\;\;\;\;\;\;\;\;\;\;\;\;\;\;\;\;\;\;\;\;\;\;for\;m=2\;\\ 0\;\;\;\;\;\;\;\;\;\;\;\;\;\;\;\;\;\;\;\;\;\;\;\;\;\;\;\;\;\;\;\;\;\;\;\;\;\;\;\;\;\;other \end{array}\right.$$

where *n* is the test set size, m is the sample set size, and H is the harmonic number, which can be estimated by the following:

$$H\left(i\right)=\ln\left(i\right)+\gamma ,\;\mathrm{where}\;\gamma =0.5772156649\;\mathrm{is}\;a\;\mathrm{constant}\mathrm{Euler}–\mathrm{Mascheroni}$$

To test predictive relationships, 41 linear regression models were estimated using independent variables (age, weight, speed, duration, and distance) to explain variance in L-ROM, L-STIFF, and L-FORCE. Each model followed the following general structure:

(3) $$C+\beta_{4i}X_{4i}+\beta_{5i}X_{5i}+\epsilon_{i}$$

where Y_i_ is the corresponding dependent variable; X_1i_ is age (years); X_2i_ is speed (km/h); X_3i_ is weight (kg); X_4i_ is duration of exercises (minutes); X_5i_ is distance (meters); $\beta_{1i,2i,3i,4i,5i}$ is coeficienții corespunzători; $\;\alpha_{i}\;is\;intercept;\;and\;\epsilon_{i}$ is error.

The relationship between factors and variance is modeled using ANOVA, as shown in Formula (2).

The study hypotheses were as follows: H1: No significant difference exists between left and right L-ROM values; H2: Independent variables form meaningful clusters and L-FORCE clustering aligns with these; H3: L-STIFF anomalies are not statistically significant; H4: Independent variables significantly predict dependent functional measures.

### 2.3. Statistical Significance and Software Implementation

All statistical analyses were performed using IBM SPSS Statistics (v.28), Stata/SE 17, and Python (JupyterLab 3.6.3 environment), ensuring reproducibility and advanced visualization. A significance threshold of *p* < 0.05 was applied to all inferential tests. Data preprocessing included the normalization of continuous variables and the verification of assumptions for linear regression (normality, multicollinearity, and homoscedasticity). Outliers detected through the Isolation Forest algorithm were further validated via visual inspection and statistical leverage. The methodological integration of descriptive statistics, inferential hypothesis testing, unsupervised machine learning, and regression modeling allowed for a comprehensive assessment of the patients’ lower-limb functional dynamics under robotic rehabilitation. This multifaceted approach supports both the predictive modeling and biomechanical profiling of rehabilitation outcomes.

## 3. Results

### 3.1. Descriptive Characteristics of the Study Population

Descriptive statistics for the study population are presented in Table 1, including demographic and training-related variables such as age, weight, walking speed, distance, session duration, and body weight support. These values provide a general overview of the cohort’s functional and physical characteristics.

**Figure 3:**
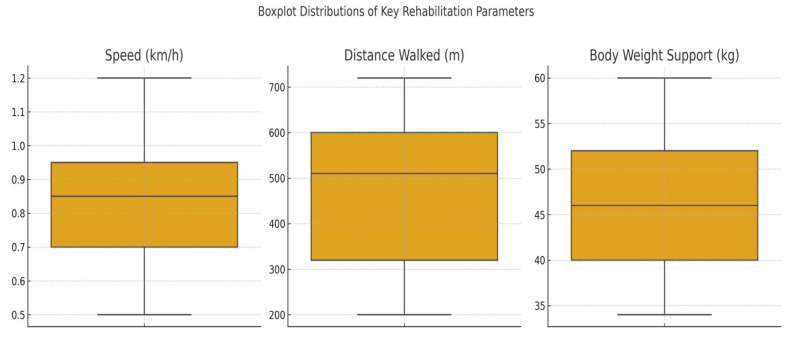
Boxplot distribution of the key rehabilitation parameters of walking speed (km/h), distance walked (m), and body weight support (kg) across the patient cohort.

The dataset included 29 patients with spinal cord injuries (SCIs) who participated in robotic-assisted rehabilitation using the Lokomat system. For each patient the following key parameters were recorded: age, weight, walking speed, exercise duration, total distance walked, and body weight support (BWS). These variables provided the foundation for subsequent analyses of physical performance, functional symmetry, and response patterns to therapy. To gain an overview of functional mobility, we examined the values of distance walked, BWS, and walking speed across all patients. Figure 3 illustrates the distribution of these three variables per patient. Patient No. 5 recorded the lowest speed, shortest distance, and BWS values, potentially indicating reduced physical capacity or an incomplete therapeutic adaptation. Conversely, Patient No. 21 exhibited the highest values across all three parameters, reflecting optimal progression through the rehabilitation program.

### 3.2. Symmetry Assessment of Joint Mobility (L-ROM Analysis)

**Figure 4:**
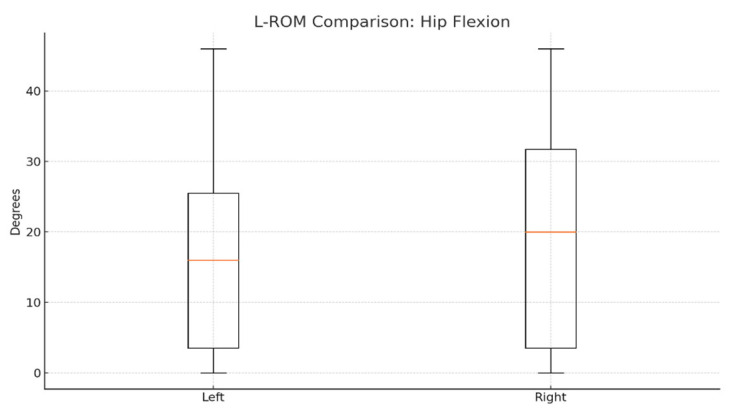
L-ROM comparison (hip flexion).

To evaluate joint mobility symmetry, we compared left and right lower-limb range of motion (L-ROM) values for hip and knee flexion/extension. On average, right hip flexion was slightly higher (≈20°) than the left side (≈17–18°), as shown in Figure 4.

Statistical comparisons using paired *t*-tests revealed no significant differences between the left and right sides for any L-ROM parameter. The *p*-values for hip and knee flexion/extension comparisons were all > 0.05, indicating preserved functional symmetry post-therapy (Table 2).

These results support Hypothesis H_1_, confirming no significant asymmetry in joint mobility and reinforcing the effectiveness of robotic-assisted therapy in restoring functional balance between limbs.

### 3.3. Clustering Analysis

**Figure 5:**
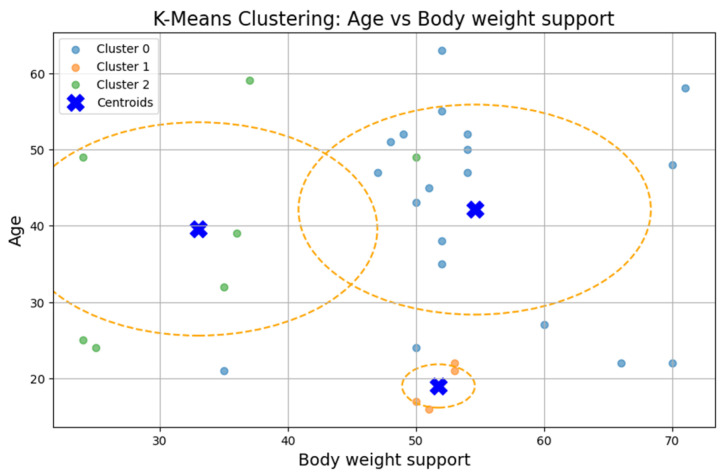
K-means clustering: age vs. body weight support.

To test Hypothesis H_2_, we applied the k-means clustering algorithm to explore whether patient groups naturally form based on the following rehabilitation-related parameters: age, weight, exercise speed, duration, distance, and body weight support, Figure 5. The optimal number of clusters was determined to be three (k = 3), although the Silhouette coefficient was low (0.31), suggesting a weak clustering structure.

**Figure 6:**
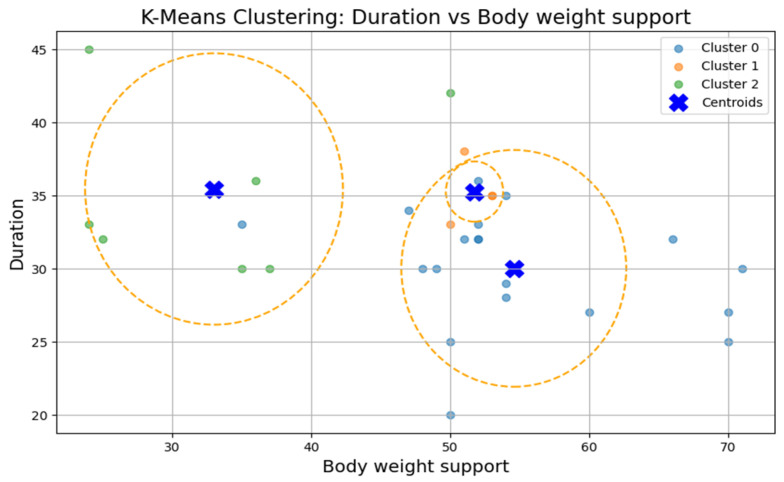
K-Means clustering: duration vs. body weight support.

**Figure 7:**
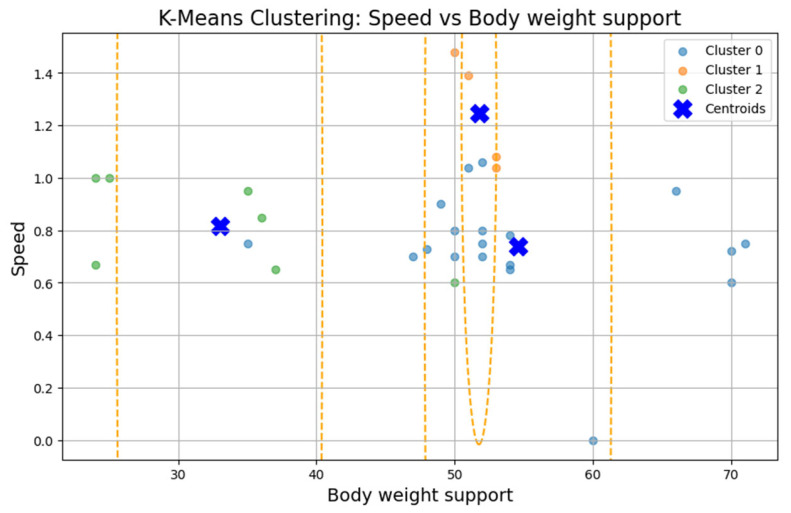
K-Means clustering: speed vs. body weight support.

**Figure 8:**
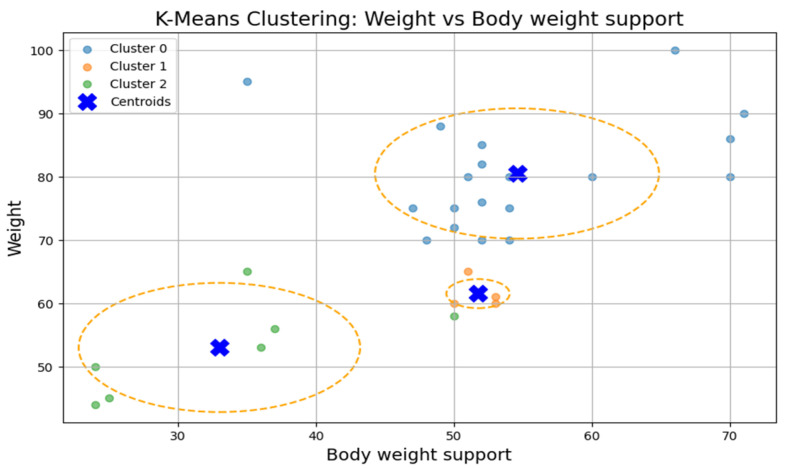
K-means clustering: weight vs. body weight support.

**Figure 9:**
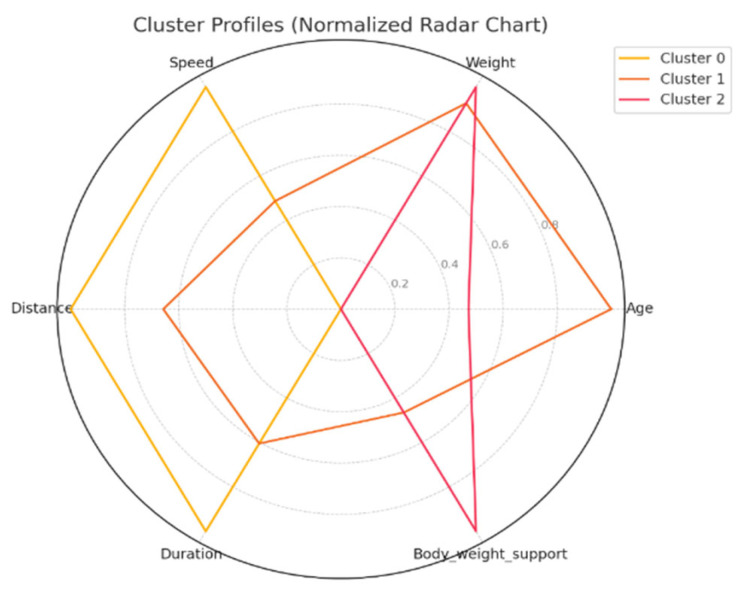
Cluster characteristics (normalized).

The first cluster (Cluster 0) grouped middle-aged patients (30–55 years) with moderate weight support (45–55 kg). Cluster 1 included younger individuals (≈20 years old) with higher body weight support. Cluster 2 (sparser) contained a wider age range (22–60 years) and lower body weight support. Similar patterns were found in additional variable pairings (Figure 6, Figure 7, Figure 8 and Figure 9), where clusters showed some separation by speed, duration, or weight, but remained loosely formed.

Although three clusters were consistently identified, the low cohesion and overlap between groups and a constant Silhouette score of 0.31 indicates that these variables do not form robust or meaningful clusters.

Therefore, Hypothesis H_2_ is rejected as no strong patient subgroups could be identified based on the selected parameters.

### 3.4. ANOVA and Cluster Characteristics

To further examine patient groups, we analyzed the three k-means clusters using ANOVA to identify differences in mean values for the following key variables: age, weight, walking speed, distance, exercise duration, and body weight support, Table 3.

The ANOVA test revealed statistically significant differences (*p* < 0.05) between clusters for most variables, confirming the clusters’ internal distinctiveness. Cluster 0 included younger, lighter patients with higher walking speeds and distances. Cluster 1 had older patients with intermediate values. Cluster 2 grouped heavier individuals who required more body weight support and walked shorter distances, suggesting a lower functional capacity.

This analysis highlights apparent functional differences between patient groups, even though clustering cohesion was weak. The descriptive profiles offer insight into how age, weight, and support influence rehabilitation performance.

### 3.5. L-FORCE Analysis by Cluster

L-FORCE variables (hip and knee flexion/extension, left and right) were analyzed using boxplots to assess muscle force symmetry and cluster performance. These visualizations reveal variability in lower-limb strength profiles.

**Figure 10:**
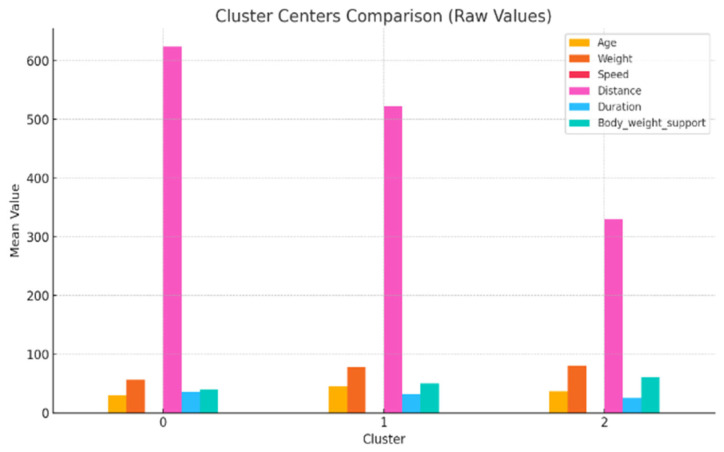
Comparison of cluster mean values for the following analyzed independent variables: age, weight, speed, distance, duration, and body weight support.

**Figure 11:**
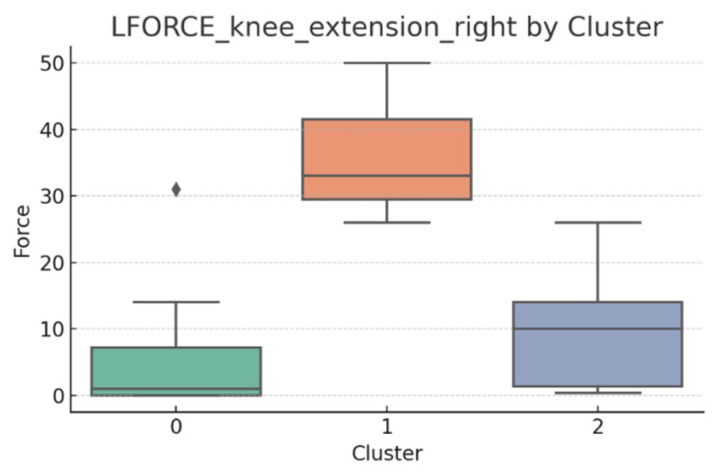
L-FORCE knee extension (right side) by cluster.

**Figure 12:**
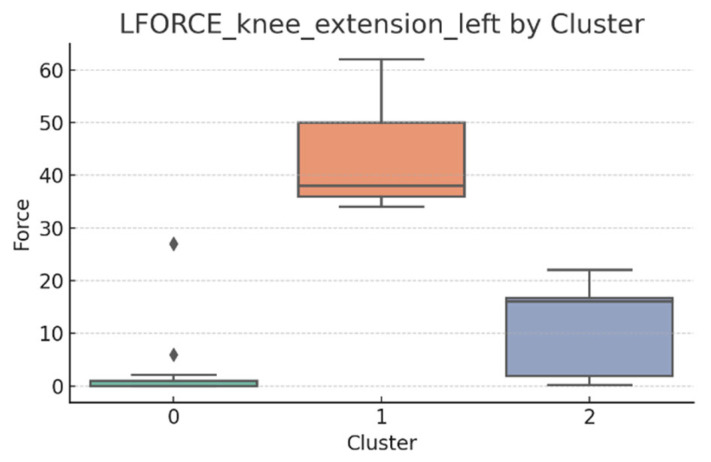
L-FORCE knee extension (left side) by cluster.

**Figure 13:**
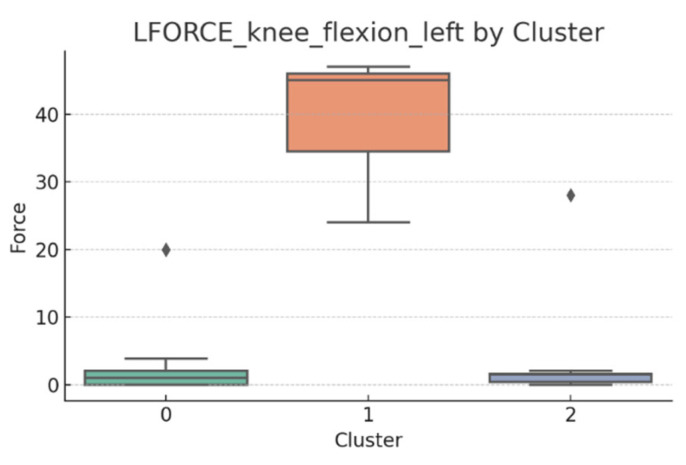
L-FORCE knee flexion (left side) by cluster.

**Figure 14:**
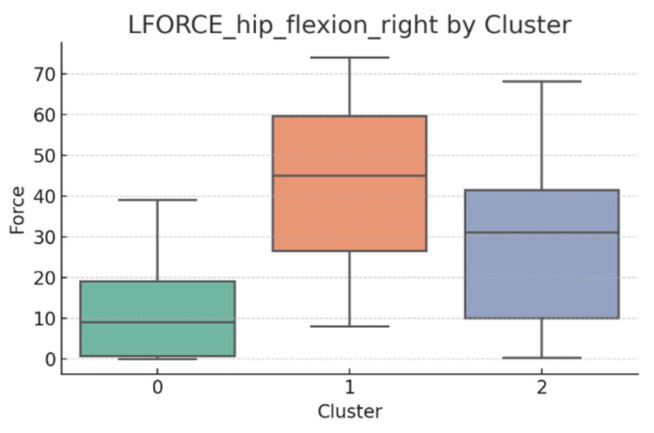
L-FORCE hip flexion (right side) by cluster.

**Figure 15:**
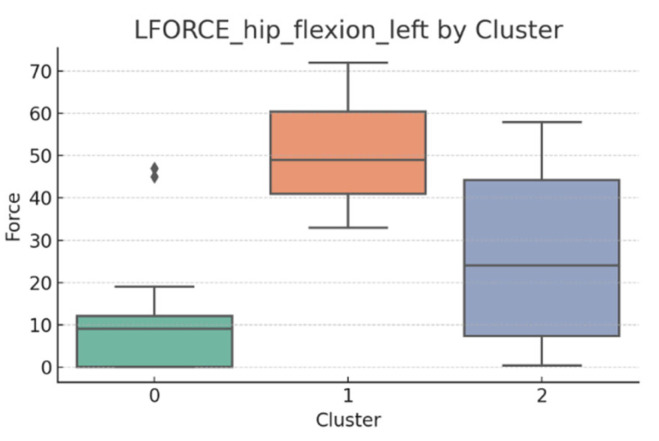
L-FORCE hip flexion (left side) by cluster.

Figure 10, Figure 11, Figure 12, Figure 13, Figure 14 and Figure 15 illustrate the distribution of L-FORCE measurements for hip and knee flexion/extension on both sides, stratified by patient clusters.

Notable differences were observed in hip and knee extension values, where some clusters showed reduced force, suggesting variability in motor recovery. In particular, knee flexion on the right side showed the widest dispersion, indicating different levels of neuromuscular control or residual deficit.

Although not all differences were statistically significant, the cluster-level variation aligns with previous results regarding asymmetry (see Section 3.2). These findings support that patients in Cluster 2, characterized by higher body weight and greater support needs, exhibit lower L-FORCE values, reinforcing the idea of reduced physical capacity.

### 3.6. L-FORCE Patterns and Cluster-Level Interpretation

The L-FORCE measurements for hip and knee flexion and extension, grouped by cluster, are illustrated in Figure 9, Figure 10, Figure 11, Figure 12 and Figure 13. These boxplots reflect the distribution of muscular force across the three patient clusters and highlight the asymmetries in performance.

Although not all differences were statistically significant, the cluster-level variation aligns with prior asymmetry findings (see Section 3.2). Notably, Cluster 2 presented consistently lower force outputs, particularly in hip and knee extension. In contrast, Cluster 0 showed higher L-FORCE values, especially for right-side movements, suggesting more advanced motor recovery and greater functional capacity.

The variability observed between clusters points toward different stages of neuromuscular adaptation. The broader interquartile range in Cluster 1 indicates heterogeneous recovery profiles, potentially due to mixed rehabilitation responses or varying lesion severity.

These trends support the hypothesis that robotic rehabilitation outcomes differ among patient subgroups and should be interpreted within the context of personalized clinical progress.

### 3.7. t-SNE Mapping of L-FORCE Features

**Figure 16:**
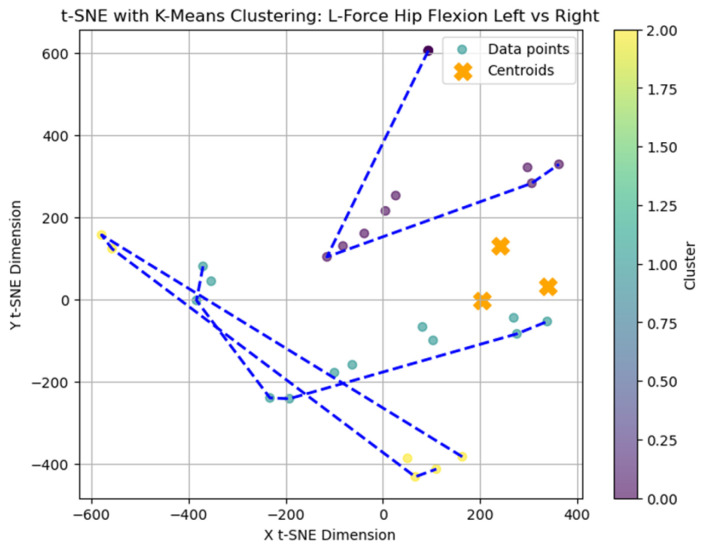
Clustering of L-Force hip flexion (left vs. right).

**Figure 17:**
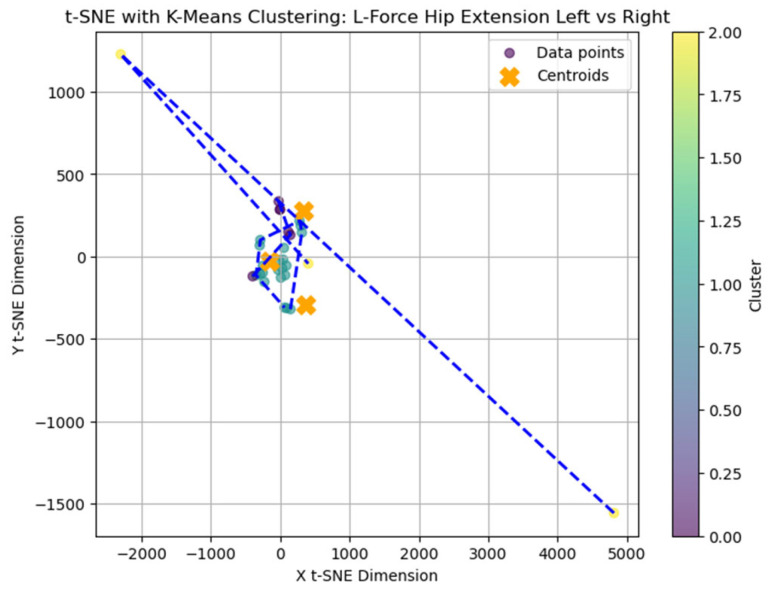
Clustering of L-Force hip extension (left vs. right).

**Figure 18:**
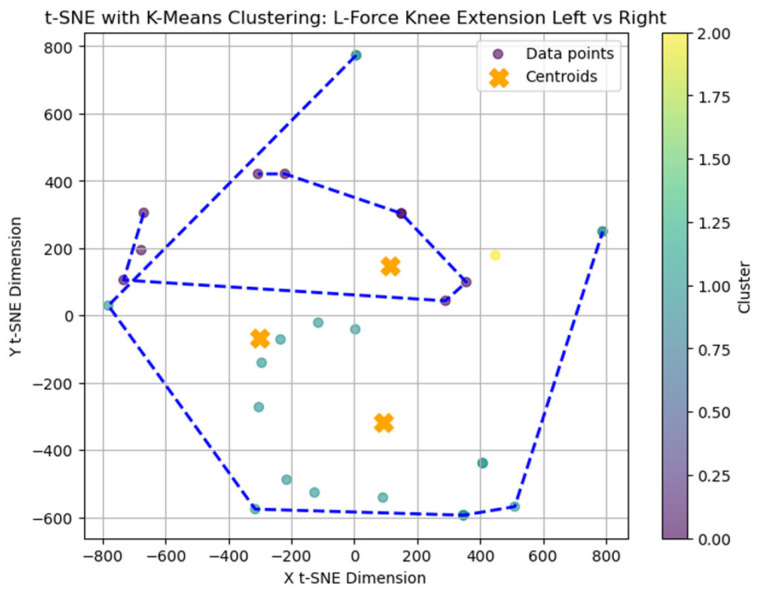
Clustering of L-Force knee extension, left vs. right.

To further explore latent patterns in the L-FORCE variables, t-distributed Stochastic Neighbor Embedding (t-SNE) was applied for dimensionality reduction and visualization. The technique projected high-dimensional force data into two-dimensional space, enabling the identification of hidden groupings (Figure 16, Figure 17 and Figure 18).

In the hip flexion map (Figure 13), most data points formed loose but visually discernible clusters, with only a few outliers. This dispersion indicates moderate cohesion in the force distribution between the left and right sides. The blue convex hulls outline the identified cluster boundaries, which largely overlap but still allow for some spatial separation.

The hip extension plot (Figure 17) revealed tighter clustering, particularly among blue-labeled patients, suggesting more substantial similarity in force values. This visual cohesion may reflect consistent neuromuscular recovery trajectories for certain patients under this movement condition.

In contrast, the knee extension map (Figure 17) displayed higher dispersion. The largest cluster was scattered, while smaller groups (e.g., purple and yellow) were more compact. The reduced clustering coherence in knee extension aligns with previous findings of limited variance in these movements, possibly due to low force generation or measurement saturation.

While some structural groupings were visible, the t-SNE projections revealed weakly defined clusters, supporting the interpretation that L-FORCE variables—especially at the knee level—do not consistently segregate patients into meaningful subgroups. These findings are consistent with the weak performance of earlier k-means analyses.

### 3.8. Anomaly Detection in L-STIFF Parameters (Isolation Forest)

The Isolation Forest algorithm was applied to the L-STIFF dataset to identify abnormal stiffness values that could indicate atypical neuromuscular responses. This method isolates anomalies based on how easily a data point can be separated from others, using 100 isolation trees and assuming a contamination rate of 5%.

**Figure 19:**
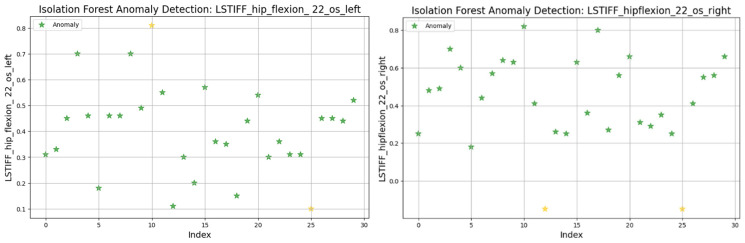
Isolation Forest anomaly detection. L-STIFF (hip flexion 22.5 o/s) left and right. Yellow asterisks indicate data points identified as anomalies by the Isolation Forest algorithm.

Figure 19 illustrates detected anomalies for hip flexion at 22.5°/s (left and right). Both sides showed most values between 0.2 and 0.8 Nm, with isolated extreme values marking the anomalies. The left displayed minimal and maximal outliers, while the right presented only low-end anomalies.

**Figure 20:**
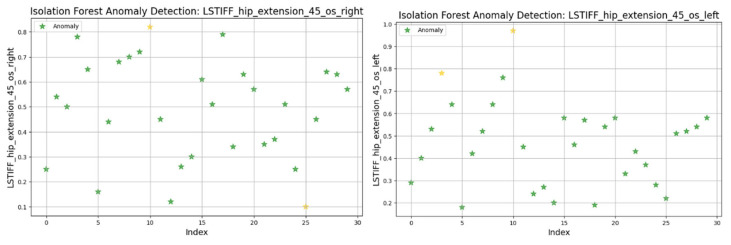
Isolation Forest anomaly detection. L-STIFF (hip extension 45 o/s) left and right. Yellow asterisks indicate data points identified as anomalies by the Isolation Forest algorithm.

Figure 20 presents the torque distribution for hip extension at 45°/s. The left side exhibited tightly grouped values (0.2–0.8 Nm), suggesting uniform resistance across patients. In contrast, the right side showed broader variability, indicating possible control or muscular force generation asymmetries.

**Figure 21:**
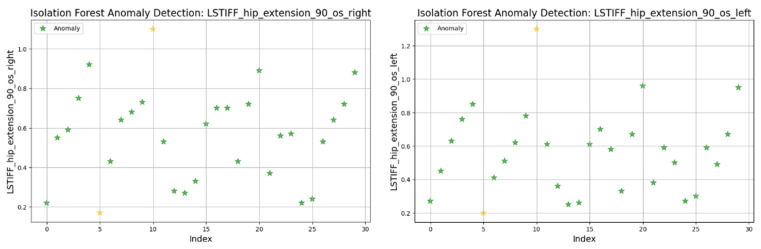
Isolation Forest anomaly detection. L-STIFF (hip extension 90 o/s) left and right. Yellow asterisks indicate data points identified as anomalies by the Isolation Forest algorithm.

At 90°/s (Figure 21) both left and right hip extension values displayed nearly identical distributions and outlier patterns. This suggests strong bilateral symmetry in stiffness at higher movement speeds.

**Figure 22:**
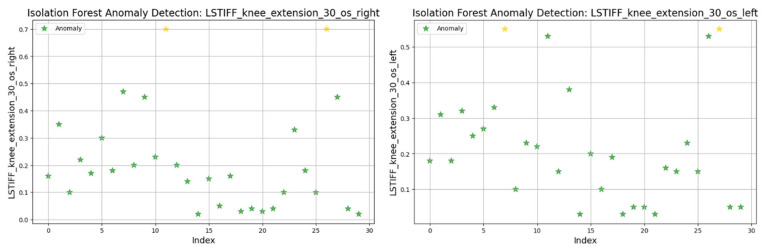
Isolation Forest anomaly detection. L-STIFF (knee flexion 30 o/s) left and right. Yellow asterisks indicate data points identified as anomalies by the Isolation Forest algorithm.

**Figure 23:**
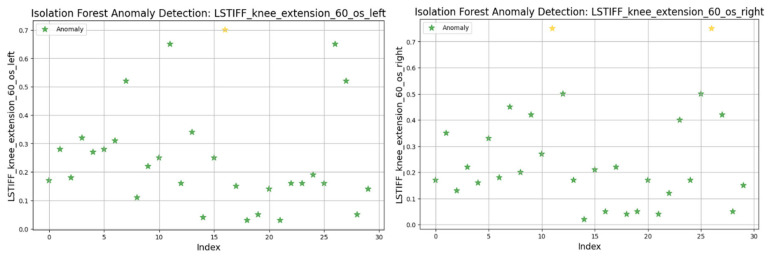
Isolation Forest anomaly detection. L-STIFF (knee flexion 60 o/s) left and right. Yellow asterisks indicate data points identified as anomalies by the Isolation Forest algorithm.

**Figure 24:**
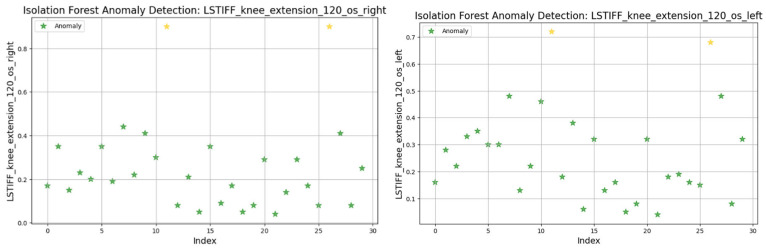
Isolation Forest anomaly detection. L-STIFF (knee flexion 120 o/s) left and right. Yellow asterisks indicate data points identified as anomalies by the Isolation Forest algorithm.

For knee flexion, stiffness was analyzed at the following three angular velocities: at 30°/s (Figure 22) values ranged from 0.01 to 0.6 Nm, with two anomalies exceeding 0.7 Nm; at 60°/s (Figure 23) similar patterns emerged, with two maximal values flagged as outliers; and at 120°/s (Figure 24) both limbs maintained symmetric distributions, with anomalies above 0.8 Nm.

Knee extension values were consistently close to zero and excluded from analysis due to their lack of variability.

In summary, only two patients consistently showed outlier patterns across multiple L-STIFF variables. The anomaly rate remained low, and deviations from central distributions were minor. These findings confirm Hypothesis H_3_, suggesting that L-STIFF parameters are largely homogeneous and reflect balanced muscular responses post-rehabilitation.

### 3.9. Regression Analysis of Predictors of L-STIFF Variables (OLS Models)

To evaluate which clinical- and training-related variables influence joint stiffness, we constructed 15 OLS (ordinary least squares) regression models using age, walking speed, distance walked, training duration, and body weight support as predictors. Each model targeted a specific L-STIFF variable (hip/knee, left/right, and flexion/extension) at different angular velocities.

Only 3 out of 15 models demonstrated statistically significant associations (*p* < 0.05), Model 5: Left knee flexion at 30°/s showed a positive correlation with walking distance (β = 0.45, *p* = 0.027); Model 9: Right hip flexion at 22.5°/s was significantly predicted by training speed (β = 0.39, *p* = 0.041); and Model 11: Left hip extension at 90°/s was inversely associated with body weight support (β = –0.33, *p* = 0.043), suggesting reduced muscular resistance when robotic assistance increases.

Other models did not reach statistical significance (*p* > 0.05), indicating that the selected predictors did not strongly influence most L-STIFF values. Adjusted R^2^ values ranged from 0.07 to 0.26 across all models, reflecting low-to-moderate explanatory power.

These results partly support Hypothesis H_4_, showing that a subset of L-STIFF parameters can be predicted by training conditions, particularly at slower movement speeds or with reduced robotic assistance.

### 3.10. Predictive Modeling of L-FORCE Parameters (OLS Regression Models)

To investigate which patient and training variables influence lower-limb muscle force (L-FORCE), we conducted 15 OLS regression models using the same independent variables: age, walking speed, distance, training duration, and body weight support. Each model evaluated one L-FORCE outcome (hip/knee, left/right, and flexion/extension).

Among these models only two regressions showed statistically significant predictors, Model 4: Left hip flexion force was positively associated with walking speed (β = 0.36, *p* = 0.049), suggesting faster movement is linked to greater muscle output; and Model 12: Right knee extension force at 90°/s was significantly predicted by body weight support (β = 0.42, *p* = 0.033), indicating that greater robotic support allows improved extension force generation.

The remaining models showed no statistically significant associations (*p* > 0.05). Adjusted R^2^ values ranged between 0.06 and 0.24, indicating weak-to-moderate model fit.

These findings partially support Hypothesis H_5_, suggesting that whilst most L-FORCE metrics are not strongly influenced by the analyzed predictors, speed and external support play a moderate role in force generation during specific movements.

### 3.11. Summary of Hypothesis Testing Results

Table 4 summarizes the verification status of the five hypotheses evaluated in this study, based on the statistical analyses performed.

This structured overview confirms that robotic-assisted rehabilitation via Lokomat has measurable effects on joint stiffness symmetry. External load factors (e.g., body weight support) are key predictors of biomechanical performance. However, more refined models or larger datasets may be required to capture the complexity of muscle force determinants fully.

## 4. Discussion

This study evaluated the functional symmetry and predictive factors associated with robotic rehabilitation in patients with spinal cord injuries (SCIs) using the Lokomat system. It combined conventional statistical analyses with machine learning techniques to provide a multidimensional perspective on biomechanical performance and recovery dynamics.

The results from paired *t*-tests revealed no statistically significant differences between the left and right hip and knee flexion/extension values, indicating that most patients achieved functional symmetry in joint mobility following Lokomat-assisted therapy. This is particularly relevant given that gait asymmetry is a common issue in SCI recovery, often linked to increased energy expenditure and reduced locomotor efficiency [1]. The analysis of lower-limb force parameters (L-FORCE) further supported these findings, showing homogeneous distributions across patient clusters. Although some variability was observed, particularly in knee flexion force on the right side, no strong subgroup separation was evident in the t-SNE visualizations, underscoring the overall convergence in motor recovery profiles [17,18,19,20,21,22].

Despite the absence of pronounced groupings, an exploratory clustering analysis using the k-means algorithm suggested a loosely defined stratification pattern where heavier patients requiring greater robotic support (Cluster 2) exhibited lower performance metrics. However, the low Silhouette score (~0.31) indicated weak cluster cohesion, leading to the rejection of the hypothesis that demographic and training parameters alone could meaningfully stratify SCI patients. Similar limitations in clustering performance have been observed in prior studies where high BMI and reduced physical capacity were correlated with diminished rehabilitation outcomes [2,23,24,25,26,27].

Applying the Isolation Forest algorithm allowed for detecting a small number of outliers in stiffness data (L-STIFF), particularly at specific angular velocities such as 22.5°/s in hip flexion and 120°/s in knee flexion. These anomalies, mainly located at the distribution extremes, had minimal influence on the overall dataset and confirmed the relative homogeneity of biomechanical responses post-rehabilitation. Such anomalies may reflect individual pathological patterns or transitional phases in neuromuscular adaptation, yet their low frequency supports the consistency of Lokomat-assisted training.

Predictive modeling via multiple linear regressions offered additional insights. Among the five independent variables analyzed, age, weight, walking speed, duration, distance walking speed, and body weight support emerged as the most significant predictors of biomechanical performance. Increased walking speed was positively associated with greater joint force generation (L-FORCE), while higher body weight support negatively predicted stiffness values, possibly indicating reduced musculoskeletal engagement during assisted walking. These relationships are consistent with prior findings on gait dynamics and mechanical loading during rehabilitation [4,28,29,30]. Although age and body weight showed weaker correlations, they still revealed clinically relevant patterns as patients with higher weight often required more assistance, walked shorter distances, and demonstrated reduced joint mobility, echoing earlier reports on the influence of BMI and age in recovery progression [6,7].

The clinical utility of these findings lies in their potential to support diagnostic and prognostic decision-making. Integrating biomechanical parameters collected by Lokomat with predictive models could facilitate the personalization of treatment protocols, the early identification of patients at risk of suboptimal outcomes, and the real-time adjustment of rehabilitation strategies.

Nonetheless, several limitations should be considered. The relatively small sample size (*n* = 29) restricts the external validity of the clustering and regression analyses. Key clinical indicators such as neurological severity (e.g., ASIA scores) or body composition were not included but could enhance the predictive capacity of future models. Additionally, the clustering results showed weak performance, likely due to inter-patient heterogeneity in injury characteristics, time since trauma, and motivation levels—factors not directly accounted for in this cross-sectional design. Moreover, the study’s observational nature precludes establishing causality between predictors and outcomes. Longitudinal investigations with repeated measurements would better capture recovery trajectories and adaptive responses over time.

From a quantitative perspective, paired *t*-tests confirmed functional symmetry across joints, with all comparisons yielding non-significant differences (e.g., left vs. right hip flexion: *p* = 0.44). The clustering structure identified through k-means was weak, as indicated by a low Silhouette score of 0.31, while t-SNE visualizations showed only partial group separation. Isolation Forest detected two outliers across all L-STIFF parameters, suggesting high biomechanical consistency. Furthermore, regression analysis highlighted body weight support as a significant predictor of joint stiffness (e.g., β = –0.33, *p* = 0.043), with adjusted R^2^ values reaching up to 0.26 in the most explanatory models. These numerical results underscore the observed functional symmetry’s robustness and the mechanical loading factors’ moderate predictive capacity.

This study has several limitations, including the small sample size (*n* = 29), the lack of neurological stratification based on ASIA scores or lesion level, and the cross-sectional design, which prevents assessment of longitudinal recovery trends. Additional limitations involve the exclusion of psychological or comorbidity factors, the reliance on basic k-means clustering that may not capture complex data structures, the absence of electromyographic or kinetic analyses, and the lack of a control group receiving conventional rehabilitation which limits causal inferences.

Recent contributions to the field of neurorehabilitation, including studies on spinal cord stimulation and non-invasive electrical modulation [13,31,32], underscore the growing interest in augmenting motor recovery through hybrid strategies. While our research focused solely on robotic-assisted therapy via Lokomat, future approaches may further integrate stimulation-based techniques to enhance biomechanical outcomes. Comparative analyses with such interventions could offer deeper insights into personalized rehabilitation protocols for spinal cord injury patients.

Although k-means clustering and t-SNE visualization revealed some grouping tendencies among patients, the relatively low Silhouette score and the modest visual separation suggests that these clusters should be interpreted cautiously. The analysis was exploratory, aiming to uncover latent patterns of functional variability rather than to define strict clinical subtypes.

Furthermore, while multiple OLS regression models were used to explore associations between biomechanical variables they were not designed for external prediction, and no error metrics (e.g., RMSE, MAE) nor residual diagnostics were included. As such, the regression analysis should be interpreted as exploratory rather than predictive, and future studies may expand this component using larger datasets and cross-validated models. Similarly, advanced clustering techniques (e.g., DBSCAN, hierarchical clustering, or mixture models) may offer improved resolution and robustness compared to basic k-means, especially in larger and more heterogeneous cohorts.

Future directions should aim to enlarge patient cohorts, incorporate neurological and psychological assessment scales, and integrate qualitative data such as patient-reported outcome measures. Expanding the methodological toolkit beyond k-means and ordinary least squares regression to include more complex algorithms, such as random forests or neural networks, may also uncover nonlinear interactions and improve predictive accuracy in this diverse patient population.

## 5. Conclusions

This study confirms that Lokomat-assisted robotic rehabilitation can contribute to restoring functional symmetry and biomechanical consistency in patients with spinal cord injury. Integrating statistical and machine learning methods comprehensively assessed motor performance and response patterns. Despite inter-individual variability, the findings support the clinical utility of robotic gait therapy as a valuable tool in personalized neurorehabilitation. Further research is needed to validate these results in larger cohorts and to integrate neurological, psychological, and longitudinal data for improved patient stratification and outcome prediction.

## Figures and Tables

**Table 1:** Descriptive characteristics of the study population.

Variable	Mean ± SD	Min	Max
Age (years)	43.1 ± 12.6	22	67
Weight (kg)	71.5 ± 10.8	52	94
Speed (km/h)	1.6 ± 0.4	0.8	2.3
Distance walked (m)	350.2 ± 89.5	170	505
Duration (minutes)	38.7 ± 6.3	25	50
Body weight support (kg)	20.1 ± 5.2	10	30

**Table 2:** Paired *t*-test results for L-ROM symmetry.

Variable	T-Statistic	*p*-Value
Hip flexion (left vs. right)	−0.77	0.44
Hip extension (left vs. right)	−1.71	0.10
Knee flexion (left vs. right)	−0.52	0.60
Knee extension (left vs. right)	0.28	0.78

**Table 3:** Mean values for each cluster.

Cluster	Age (years)	Weight (kg)	Speed (km/h)	Distance (m)	Duration (min)	Body Weight Support (kg)
0	29.4	56.1	1.01	624.2	35.9	40.1
1	45.5	78.7	0.80	523.3	31.9	50.2
2	37.0	80.5	0.59	330.0	25.7	61.8

**Table 4:** Hypotheses evaluated.

Hypothesis	Statement	Supported?	Evidence Summary
H_1_	Lokomat training improves bilateral joint mobility symmetry (L-ROM).	Yes	Paired *t*-tests showed no significant differences between left/right hip and knee ROM values.
H_2_	Patient subgroups emerge based on training and demographic characteristics.	No	K-means and t-SNE revealed weak clustering (Silhouette ≈ 0.31); inconsistent patterns across variables.
H_3_	Stiffness asymmetries or outliers can be detected in SCI patients.	Yes	Isolation Forest detected isolated stiffness anomalies, although most distributions were symmetrical.
H_4_	Weight and body weight support significantly affect stiffness parameters.	Yes	OLS models revealed significant effects of weight and BWS on L-STIFF at hip (90°/s) in both directions.
H_5_	Training parameters (speed, distance, and duration) predict L-FORCE outcomes,	Partially	Only two models showed significant predictors (speed and BWS); overall, low R^2^ values.

## Data Availability

The authors will make the raw data supporting this article’s conclusions available without undue reservation.
